# Vibration Characteristic Analysis of Hollow Fiber Membrane for Air Dehumidification Using Fluid-Structure Interaction

**DOI:** 10.3390/membranes13020233

**Published:** 2023-02-15

**Authors:** Caihang Liang, Jiaxing Chen, Nanfeng Li, Yanfang Dong, Tao Zhong, Si Zeng, Chuanshuai Dong

**Affiliations:** 1 Key Laboratory of Microelectronic Packaging and Assembly Technology of Guangxi Department of Education, School of Mechanical and Electrical Engineering, Guilin University of Electronic Technology, Guilin 541004, China; 2 Guangdong Provincial Key Laboratory of Distributed Energy Systems, Dongguan University of Technology, Dongguan 523808, China; 3 School of Energy and Built Environment, Guilin University of Aerospace Technology, Guilin 541004, China; 4 Department of Science and Technology, Nanning College for Vocational Technology, Nanning 530008, China; 5 Guangxi Beitou Environmental Protection & Water Group Co., Ltd., Nanning 530025, China; 6 Key Laboratory of Enhanced Heat Transfer and Energy Conservation of Education Ministry, School of Chemistry and Chemical Engineering, South China University of Technology, Guangzhou 510641, China

**Keywords:** hollow fiber membrane, fluid-structure interaction, modal, vibration

## Abstract

Hollow fiber membrane dehumidification is an effective and economical method of air dehumidification. The hollow fiber membrane module is the critical component of the dehumidification system, which is formed by an arrangement of several hollow fiber membranes. The air stream crosses over the fiber bundles when air dehumidification is performed. The fibers vibrate with the airflow. To investigate the characteristics of the fluid-induced vibration of the hollow fiber membrane, the two-way fluid-structure interaction model under the air-induced condition was established and verified by experiments. The effect of length and air velocity on the vibration and modal of a single hollow fiber membrane was studied, as well as the flow characteristics using the numerical simulation method. The results indicated that the hollow fiber membrane was mainly vibrated by fluid impact in the direction of the airflow. When the air velocity was 1.5 m/s~6 m/s and the membrane length was 100~400 mm, the natural frequency of the membrane was negatively correlated with length and positively correlated with air velocity. Natural frequencies were more sensitive to changes in length than changes in air velocity. The maximum equivalent stress and total deformation increased with air velocity and length. The maximum equivalent stress was concentrated at both ends, and the maximum deformation occurred in the middle. The research results provided a basis for the structural design of hollow fiber membranes under flow-induced vibration conditions.

## 1. Introduction

People in modern society spend most of their time indoors. Therefore, there is an increasing concern for indoor air quality (IAQ) in houses and offices. Especially with the outbreak of the novel coronavirus in the spring of 2020, people are more aware of IAQ. Indoor air humidity is one of the most important factors affecting IAQ. High or low indoor air humidity can harm human health and comfort. The recommended relative humidity range is 40–60% in dwellings and air-conditioned buildings in summer [1]. During summer, fresh air relative humidity is very high, and sometimes up to 90% in a coastal area. Therefore, it is necessary to dehumidify the fresh air and then supply it to the room. Fresh air dehumidification consumes a large amount of energy, which makes up 20% to 40% of the energy consumed by air conditioning systems [2,3]. The independent temperature and humidity control system can significantly decrease the energy consumption of all fresh air conditioning systems [4,5,6]. The independent temperature and humidity control system can separately handle air sensible heat and latent heat, which can significantly reduce the energy consumption of the air conditioning system. It is one of the key techniques to achieve the goal of “carbon peaking and carbon neutrality” in the field of refrigeration and air conditioning.

There are various air dehumidification approaches, including condensation dehumidification, solid adsorption dehumidification, solution dehumidification, and membrane-based liquid dehumidification. Membrane-based liquid dehumidification has been gaining much attention from both academic and industrial fields because of its high efficiency, environmental friendliness, and energy savings [7,8,9]. Membrane-based liquid dehumidification uses a selective semi-permeable membrane to selectively penetrate vapor, but not liquid and other gases, to realize the dehumidification objective [10,11,12]. Flat membrane dehumidification and hollow fiber membrane dehumidification systems are very effective methods to solve the droplet-carrying problem in traditional liquid dehumidification systems. Compared with the flat membrane, the hollow fiber membrane has the advantages of high-packing density, compactness, and high efficiency, and has been widely concerned. [13,14]. Ghidossi et al. [15] developed a mass transfer model for membranes under laminar and turbulent flow conditions and analyzed the mass transfer behavior of membranes under different flow regimes. Zhang [16] studied the hollow fiber membrane humidifier component of seawater desalination systems. A simulation model was built considering the flow, and heat and mass transfer characteristics of both the solution and air sides, and related experiments were designed to validate the model. Huang et al. [17] studied the performance of elliptical hollow fiber membrane tubes in the coupled flow and heat transfer by modeling elliptical hollow fiber membrane tubes and discovered that as the elliptical semi-axis ratio (b/a) decreased, the mean friction coefficient and the Nusselt number also decreased. Fluids flowing in the short semi-axial direction have improved combined heat transfer and fluid flow properties. Ma et al. [18] investigated the effect of membrane arrangement on heat and mass transfer. The simulation results predicted the effect of fiber distance on membrane module hydrodynamics and filtration performance. Most of these studies are from the point of view of the flow field and ignore the vibration and deformation of the membrane.

Hollow fiber membranes are flexible tubes, so during operation, flow-induced vibrations are generated when airflow crosses the membrane tubes. Flow-induced vibrations transform the shape of hollow fiber membrane tubes, thus affecting fluid flow, heat and mass transfer performance, mechanical performance, and so on. Li et al. [19] studied the effectiveness of vibration in anti-fouling control of a submerged hollow fiber membrane bioreactor. Their results showed that low-frequency continuous transverse vibration is very effective for fouling control, and it is more effective than longitudinal vibration. Low et al. [20] experimentally investigated transverse vibration on hollow fiber membrane tubes to effectively reduce the external concentration polarization and increase forward osmosis. Chai et al. [21] analyzed the performance of rotational vibration in a submerged hollow fiber membrane system for the bioseparation of high-concentration yeast suspensions. Their investigation also showed that the system performance on critical flux for the rotational vibration system is insensitive to packing density. During membrane operation, the fluid-structure interaction cannot be ignored. Fluid-structure interaction has been widely used in heat exchangers [22,23,24]. The effect of fluid-structure interaction on hollow fiber membrane performance has been studied. Zamani et al. [25] studied the effect of longitudinal vibration on the antifouling properties of hollow fiber membranes by the fluid-structure interaction (FSI) method. The effect of membrane surface wall shear rate was analyzed, and it was found that dynamic shear-enhanced filtration by vibration can reduce concentration polarization and membrane contamination. Liu et al. [26] found that the FSI was effectively evaluated in terms of the effects of fiber tube material, size, and relaxation on the shear effect and flux on the membrane tube surface, and the FSI simulated fiber tube displacements show good agreement with experimentally measured fiber displacement data. Sangeetha. [27] investigated the performance of hollow fiber membranes by coupling flow characteristics with structural mechanics to study the interaction between blood flow and membrane structure, and the results showed that the convoluted corrugated fiber model would have better mechanical stability. The majority of these studies have concentrated on the effect of solid domain changes on the entire membrane module. In considering the effects of two-way fluid-structure interaction, Jeong et al. [28] analyzed the hollow fiber membrane modules used for dehumidification in pneumatic systems. The fluid flow and dehumidification characteristics were determined by the FSI method for different shapes of deformed baffles. The difference between the results of the experiment and low analysis was about 5%. Li et al. [29] developed a two-way fluid-structure interaction model for fluid-induced fiber membrane vibration, and the fluid-induced vibration could result in heat and mass transfer enhancement factors of up to 68.9% and 96.2% compared to the non-fluid-induced vibration state. Jang et al. [30] simulated the behavior of fluid and shear stresses in a membrane reactor. The results of the fluid-structure interaction analysis showed that reducing slack can increase the high membrane tension of a membrane. The two-way fluid-structure interaction better reflects the impact of fluid interactions on overall component performance during actual operation.

As mentioned above, the FSI method improves the antifouling ability as well as the heat and mass transfer ability of the hollow fiber membrane to some extent. The vibration characteristics are very important for membrane modules, affecting the mechanical properties of the membrane and process preparation of the module. Few studies have investigated the effect of fluid-structure interaction on the vibrational properties of the membrane itself. The two-way fluid-structure interaction method can provide more accurate results to guide the module design, so the vibration characteristics of hollow fiber membranes for air dehumidification using two-way fluid-structure interaction are investigated in this paper.

To explore the vibration characteristics of the hollow fiber membrane under flow-induced vibrations, this paper establishes the two-way fluid-structure interaction vibration model of the hollow fiber membrane and verifies the mathematical model by the air-induced membrane tube vibration experiment. The effects of membrane length and air velocity on vibration deformation, equivalent stress, and natural frequency of membranes are then investigated using the numerical simulation method. According to the research results of hollow fiber membrane vibration, the goal of this paper is to provide theoretical guidance for membrane module structural design.

## 2. Numerical Model

### 2.1. Fluid-Structure Interaction Model Equations

#### 2.1.1. Fluid Domain

The air is an incompressible Newtonian fluid, and the continuity equation is:

(1) $$\frac{\partial u}{\partial x}+\frac{\partial v}{\partial y}+\frac{\partial w}{\partial z}=0$$

The momentum equations are:

(2) $$\frac{\partial u}{\partial t}+u\frac{\partial u}{\partial x}+v\frac{\partial u}{\partial y}+w\frac{\partial u}{\partial z}=-\frac{1}{\rho_{a}}\frac{\partial p}{\partial x}+\upsilon \left(\frac{\partial^{2}u}{\partial x^{2}}+\frac{\partial^{2}u}{\partial y^{2}}+\frac{\partial^{2}u}{\partial z^{2}}\right)+G_{x}$$

(3) $$\frac{\partial u}{\partial t}+u\frac{\partial v}{\partial x}+v\frac{\partial v}{\partial y}+w\frac{\partial v}{\partial z}=-\frac{1}{\rho_{a}}\frac{\partial p}{\partial y}+\upsilon \left(\frac{\partial^{2}v}{\partial x^{2}}+\frac{\partial^{2}v}{\partial y^{2}}+\frac{\partial^{2}v}{\partial z^{2}}\right)+G_{y}$$

(4) $$\frac{\partial w}{\partial t}+u\frac{\partial w}{\partial x}+v\frac{\partial w}{\partial y}+w\frac{\partial w}{\partial z}=-\frac{1}{\rho_{a}}\frac{\partial p}{\partial z}+\upsilon \left(\frac{\partial^{2}w}{\partial x^{2}}+\frac{\partial^{2}w}{\partial y^{2}}+\frac{\partial^{2}w}{\partial z^{2}}\right)+G_{z}$$

where *u*, *v*, *w* represent the velocity components in the x, y, and z directions (m^2^/s), *ρ_a_* is the density of the air (kg/m^3^), *p* is the air pressure (N), *t* is time (s), *ν* is the kinematic viscosity (m^2^/s), *G* is the gravity (N), subscripts “*x*”, “*y*”, and “*z*” are the different directions, respectively.

#### 2.1.2. Solid Domain

The solid control equation is given by Newton’s second law; its equation is:

(5) $$\rho_{s}a_{s}=\nabla \cdot \sigma_{s}+f_{s}$$

where *ρ_s_* is the density of the hollow fiber membrane (kg/m^3^), *a_s_* is the acceleration vector, *σ_s_* is the stress tensor, *f_s_* is the volume force vector.

The dynamic equation of the membrane under the air-induced condition can be written in the equation: [31]

(6) $$\left[M\right]\left\{D\right\}+\left[C\right]\left\{V\right\}+\left[K\right]\left\{A\right\}=\left\{F\right\}$$

where [*M*], [*C*], [*K*] represent the mass matrix, damping matrix, and stiffness matrix, respectively; {*D*}, {*V*}, {*A*} represent the displacement vector, velocity vector, and acceleration vector, respectively; and {*F*} represents the force vector.

#### 2.1.3. Coupling Boundary Equations of the Air and Membrane Tube

In the fluid-structure interaction calculation, the displacement (*d*) and stress (*τ*) on the fluid-structure interaction interface should meet the equilibrium condition on both sides. In other words, the following equation is satisfied: [32]

(7) $$d_{f}=d_{s}$$

(8) $$n_{f}\cdot \tau_{f}=n_{s}\cdot \tau_{s}$$

where *d* is the displacement (m), *n* is the unit normal vector of stress, which is perpendicular to the interface of the fluid and solid. Subscripts “*f*”, “*s*” are the fluid domain and solid domain, respectively.

### 2.2. Geometry and Meshing

**Figure 1:**
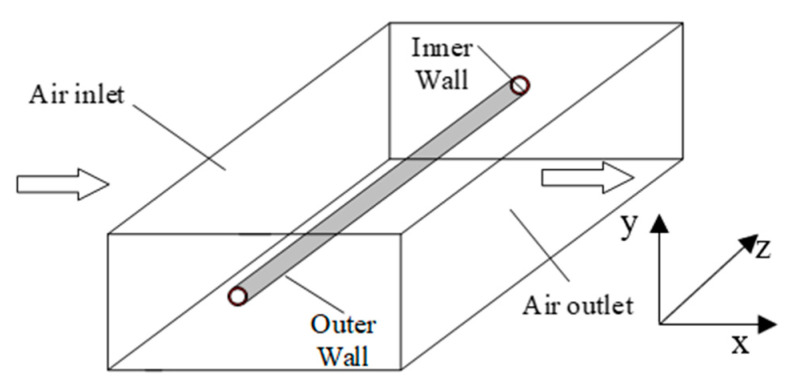
Geometric model.

**Figure 2:**
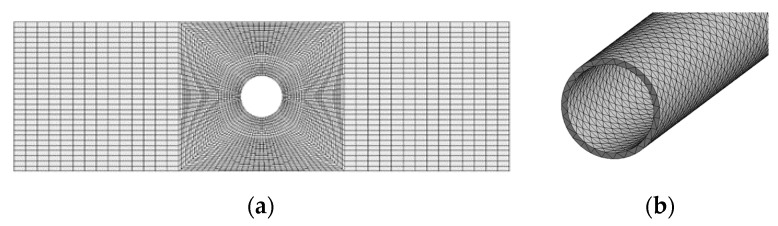
Meshing Diagram: (**a**) Fluid domain local mesh; (**b**) Solid domain local grid.

Figure 1 depicts the geometric model of a single hollow fiber membrane, and Table 1 depicts the physical parameters of the fiber membrane. After specifying the material properties, the model is meshed. The fluid domain is meshed by the 3D fluid element, with a mesh cell count of 114,000. The solid domain is made of unstructured tetrahedral mesh with a solid187 unit type. The mesh number is 87,057. The meshing is shown in Figure 2.

The mesh of the computational model directly determines the efficiency and accuracy of the computation. This paper uses the maximum deformation and maximum equivalent stress of the hollow fiber membrane as indicators to verify mesh independence. As shown in Table 2, the changes in maximum deformation and maximum equivalent stress of hollow fiber membranes with different mesh numbers (55,110, 108,300, 433,000) of 100 mm hollow fiber membranes at an air velocity of 1.5 m/s are compared. The mesh number rises from 108,300 to 433,000, and the maximum deformation and maximum equivalent stress increase is less than 0.2%. To balance the efficiency and accuracy of the calculation results, a mesh number of 108,300 is selected for solving.

### 2.3. Boundary Conditions

Considering the influence of gravity, the fluid domain adopts the velocity inlet, the air velocities are 1.5 m/s, 3 m/s, 4.5 m/s, and 6 m/s, respectively, and the corresponding Reynolds numbers are 166, 332, 498, and 664, respectively. When the air flows through the membrane tube, the fluid flow is in a transition state, and the K-epsilon turbulence model is selected [33]; the pressure outlet is set to 0 Pa; the non-slip wall is set; The SIMPLE algorithm is chosen by the velocity-pressure coupling; the interface is set to FSI, and the dynamic mesh model of the diffusion smoothing method is selected; The ends of the membrane are set as fixed constraints in the solid domain, and the outer walls of the membrane are set as coupling surfaces.

### 2.4. Fluid-Structure Interaction Solution Process

Firstly, the fluid domain and solid domain of a single hollow fiber membrane are established and specified, respectively. Both the solid domain and fluid domain are separated into meshes. The governing equations in the fluid domain are solved using the finite volume approach, and the pressure distribution on the fluid-structure interface is obtained. The system coupling module then transfers this to the solid domain. The solid domain is applied to the solid surface through the set fluid-solid interface based on the pressure obtained from the transfer solution, and the transient dynamic analysis is performed using the finite element method to obtain the displacement of the membrane tube. Finally, the module feeds the analysis results back into the fluid domain. The fluid domain’s governing equations are solved using the new boundary conditions, and the system coupling exchange calculation is repeated until the pre-specified time limit is reached. The data transfer interface in the system coupling module exchanges fluid and solid data with a time step of 0.005 s during the fluid-structure coupling calculation, which takes one second. The effective value determines the convergence of this time step result, which is judged to be convergent when it is less than 1e−4.

### 2.5. Modal Analysis and Solution

In a two-way fluid-structure interaction modal analysis, the computational fluid dynamics (CFD) flow field calculation and finite element method (FEM) transient coupling analysis are performed first. The velocity and pressure of the fluid are obtained from the fluid domain solution, displacement and stress-strain are obtained from the solid domain solution, and coupling analysis is performed after convergence to a certain accuracy for the pre-stress modal analysis. Fixed constraints are set at both ends of the fiber membrane; the large deformation is turned on to increase the calculation accuracy, and the first six natural frequencies and modes of the hollow fiber membrane are solved.

## 3. Model Verification

**Figure 3:**
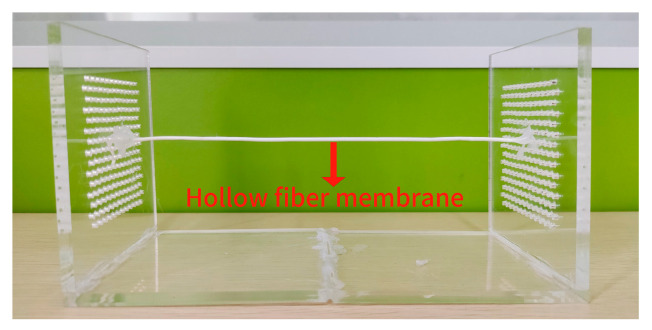
Hollow fiber membrane fixation module.

**Figure 4:**
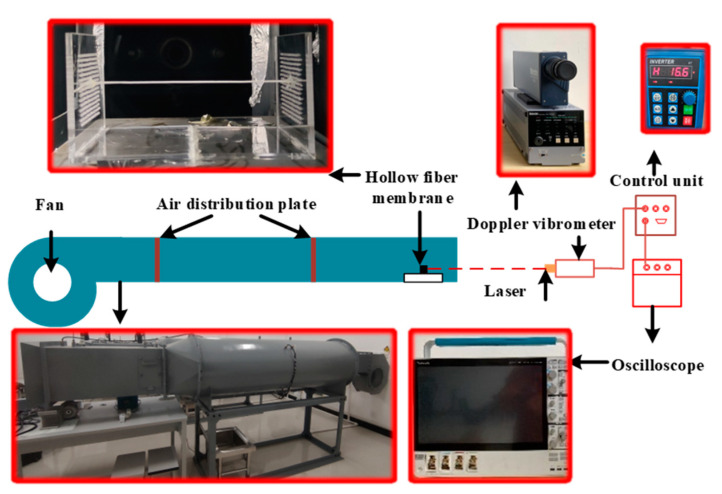
Diagram of hollow fiber membrane flow-induced vibration experimental apparatus.

The experimental validation bench was built by members of this group. Figure 3 depicts the hollow fiber membrane fixing module. Figure 4 depicts the whole experimental setup of the hollow fiber membrane flow-induced vibrations. The air velocity was controlled by a three-phase variable frequency fan (SIEMENS^®^ 1LE001, Munich, Germany), and the experimental flow channel was a 5 m long circular air duct with a built-in flow equalizing plate. The fan’s air is subjected to two rectifier flows, and the flow is fully developed when it reaches the end. At this time, it is considered that the air velocity has reached a relatively stable state. The hollow fiber membranes were then measured and observed. During the experiment, to improve the numerical accuracy of the experiment, the air intake velocity was tested in the range of 1.5~10.5 m/s. To ensure the accuracy of the air velocity measurement and reduce the experimental error, the data was collected after the fan frequency was adjusted for 60 s to stabilize it, and a hot-wire anemometer (Testo-425, Testo SE, Lake Titi, Germany) was used for collection five times at each air velocity. The uncertainty of the air velocity measurement is ±0.5%. DENSHIGIKENQ Co., Ltd.’s laser vibrometer (melectro^®^ V-100lm type-Da, DENSHIGIKENQ Co., Ltd., Shoshima City, Tokyo) was used to measure flow-induced fiber deformation (Osaka, Japan), the laser vibrometer (melectro^®^ V-100lm type-DA, DENSHIGIKENQ Co., Ltd., Shoshima City, Tokyo) can only measure vibration displacement in one plane. Since the axial direction of the hollow fiber membrane has been fixedly constrained, we only need to measure the in-plane XZ and plane YZ. After measuring the displacement vibration on a plane, we only need to adjust the placement of the fiber membrane to keep it relative to the laser vibrometer to complete the next experiment.

Table 3 shows the comparison of the results between experimental and simulated values at different air velocities. From the table, it can be seen that the maximum errors between the simulated and experimental data of membrane tube amplitude in the x and y directions are 3% and 5%, respectively. The results also show that the vibration mainly occurs in the x direction. An accurate two-way fluid-structure interaction model can be demonstrated by this. It provides a guarantee for the further simulation analysis below.

## 4. Results and Discussion

### 4.1. Effect of Length and Air Velocity on the Hollow Fiber Membrane’s Inherent Frequency and Mode Shape

At air velocities of 1.5 m/s, 3 m/s, 4.5 m/s, and 6 m/s, four hollow fiber membranes with varying lengths of 100 mm, 200 mm, 300 mm, and 400 mm, respectively, are chosen for a two-way fluid-structure interaction modal analysis. Table 4, Table 5, Table 6 and Table 7 show the first 6-order natural frequencies with different fiber lengths at different air velocities.

From Table 4, Table 5, Table 6 and Table 7, under different air velocities, the first six natural frequencies characterize the same features, decreasing with the increase in length and the speed of decrease, gradually slowing down with the decrease itself. At a 1.5 m/s air velocity, the variation in the natural frequency of each order at membrane length increasing from 100 to 200 mm is 9–12 times the variation at the membrane length ascending from 300 to 400 mm. While at an air velocity of 6 m/s, this number is 14–15 times comparing the variation in the natural frequency of each order at different increases of membrane length from 100 to 200 mm and from 300 to 400 mm, respectively. At the same time, the variation in higher order frequency with length is larger than those of lower order frequency. The increase in the length will affect the mass and stiffness of the membrane and eventually vary in natural frequency. The analyses indicate that the natural frequency is negatively correlated with the length.

Under different lengths, the first six natural frequencies characterize the same features, increasing with the increase in air velocity. The increase in air velocity will increase the shear force between the walls and lead to the rise in natural frequency. This rise is small compared to the effect of length on the natural frequency. The natural frequencies of each order at a membrane length of 100 mm at an air velocity of 6 m/s only rise by 1–3 Hz in comparison to that at a 1.5 m/s air velocity. The law increases with the increase in the length of the natural frequencies of each order. While the membrane length is 400 mm, the natural frequencies of each order at the air velocity of 6 m/s increase by 4–8 Hz compared with those at 1.5 m/s air velocity. Through analysis, we get a positive correlation between natural frequency and air velocity. Compared with air velocity variation, the natural frequency is more sensitive to length variation.

**Figure 5:**
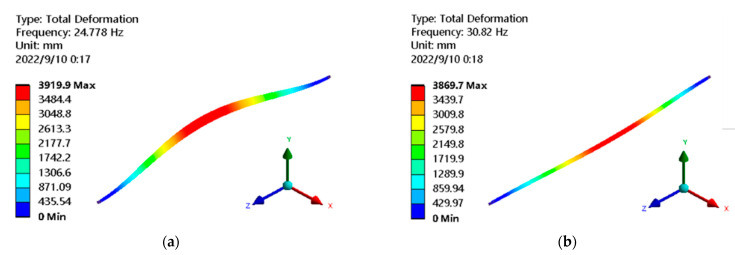

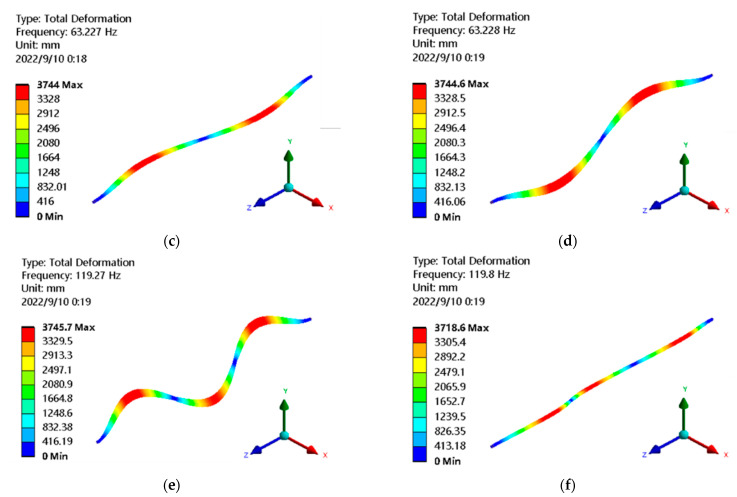
The first 6 modes of the fluid-structure interaction mode of hollow fiber membrane (length 200 mm, air velocity 3 m/s). (**a**) the first order; (**b**) the second order; (**c**); the third order; (**d**) the fourth order; (**e**) the fifth order; (**f**) the sixth order.

With the change in the length and air velocity, the mode shape region corresponding to each order natural frequency of the hollow fiber membrane does not change, but the corresponding value is changed. Therefore, the first six modes are given at different lengths and air velocities for an air velocity of 3 m/s and membrane length of 200 mm under the two-way fluid-structure interaction. As shown in Figure 5, the first two modes are closer to the resonant frequency due to their lower frequencies. Therefore, the shape is similar, and the distribution of deformation positions is consistent; due to the low frequency, which is closer to the resonant frequency, and the fact that the air flow is mainly concentrated in the middle, the vibration in the middle part is more intense. With the increase in the order, the frequency increases, distortion can easily occur in a certain range, the area of severe deformation increases, and the maximum deformation creeps from the middle to both ends. Compared to the first order, the maximum deformation of the sixth order is reduced by 5%. This demonstrates that there is a connection between deformation and order.

### 4.2. Effect of Length and Air Velocity on the Hollow Fiber Membrane Vibration

**Figure 6:**
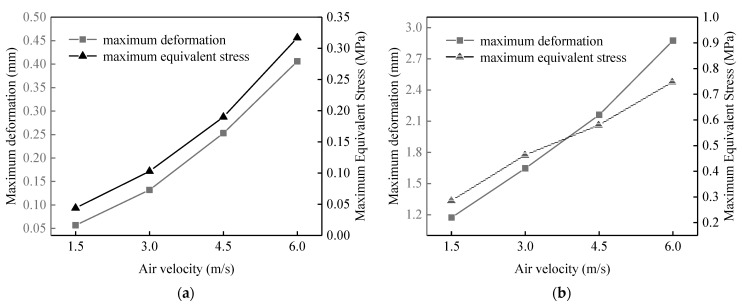

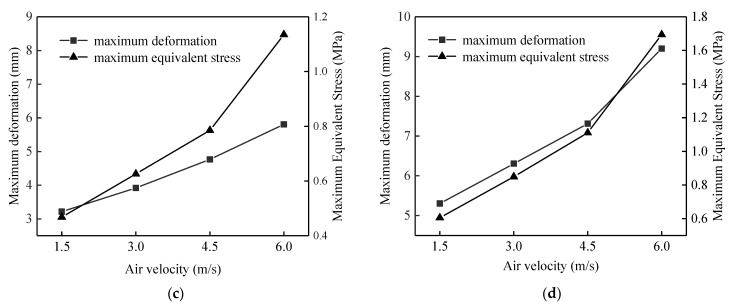
The maximum deformation and equivalent stress of fluid-structure interaction at different air velocities for different fiber lengths. (**a**) 100 mm; (**b**) 200 mm; (**c**) 300 mm; (**d**) 400 mm.

Four hollow fiber membranes with varying lengths of 100 mm, 200 mm, 300 mm, and 400 mm were chosen for a two-way fluid-structure interaction vibration study at the air velocities of 1.5 m/s, 3 m/s, 4.5 m/s, and 6 m/s. Figure 6 shows the maximum deformation and maximum equivalent stress changes of the two-way fluid-structure interaction membranes with different fiber lengths at different air velocities.

From Figure 6, under different air velocities, with increasing length, the maximum deformation and maximum equivalent stress of the fiber increase. At 1.5 m/s air velocity, the maximum deformation and maximum equivalent stress of 100 mm fiber length are 0.057 mm and 0.044 MPa, respectively. The maximum deformation and maximum equivalent stress of 400 mm fiber length are 5.304 mm and 0.605 MPa, respectively. The maximum deformation and maximum equivalent stress of 100 mm fiber length are 1% and 7% of the maximum deformation and maximum equivalent stress of 400 mm fiber length, respectively. While at 6 m/s air velocity, the maximum deformation and maximum equivalent stress of 100 mm fiber length are 0.406 mm and 0.317 MPa, respectively. At a 400 mm fiber length, the maximum deformation and maximum equivalent stress are 9.2 mm and 1.694 MPa, respectively. The maximum deformation and maximum equivalent stress of 100 mm fiber length are 4% and 18% of those of 400 mm fiber length, respectively. This indicates that the maximum deformation and maximum equivalent stress of the membrane increase with the increase in the length at different air velocities.

Under different lengths, the maximum deformation and maximum equivalent stress of the fiber increase with the increase in air velocity. At the length 100 mm, the maximum deformation and maximum equivalent stress of the fiber at 1.5 m/s air velocity are 14% and 13% of the maximum deformation and maximum equivalent stress of the fiber at a 6 m/s speed, respectively. When the length is 400 mm, the membrane’s maximum deformation and maximum equivalent stress at 1.5 m/s air velocity are, respectively, 57% and 36% of those values at 6 m/s air velocity. This indicates that as air velocity increases, the maximum deformation and maximum equivalent stress of the membrane do as well. The same law is observed for different lengths.

**Figure 7:**
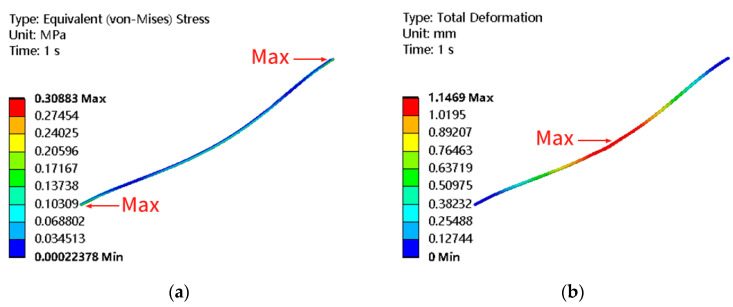
Cloud diagram of total deformation and equivalence force distribution (length 200 mm, air velocity 3 m/s). (**a**) Equivalent stress (MPa); (**b**) Total deformation (mm).

With the change in the length and air velocity, the deformation and stress regions of the membrane do not change, but the corresponding values are changed. Therefore, the total deformation and equivalent stress distribution cloud diagrams obtained by a two-way fluid-structure interaction at 3 m/s air velocity and 200 mm length of hollow fiber membrane are given under different lengths and different air velocities, as shown in Figure 7. The maximum deformation is mainly in the middle position. The maximum stress is concentrated at both ends. The fixed constraint at both ends improves the stiffness of both ends of the membrane and effectively inhibits the vibration of both ends, so the deformation amount is small but the stress is maximum. Long periods fixed at both ends are likely to lead to stress concentration as air velocity increases, which may affect the life of the membrane. Attention should be paid to the design.

### 4.3. Flow Characteristics

**Figure 8:**
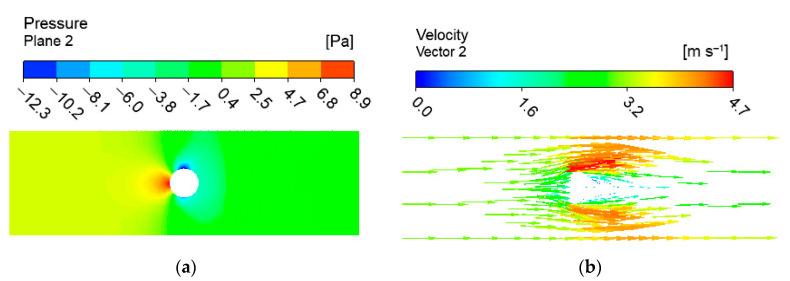
Flow field cloud diagram at z = 100 section position (length 200 mm, air velocity 3 m/s): (**a**) pressure distribution; (**b**) velocity vector distribution.

**Figure 9:**
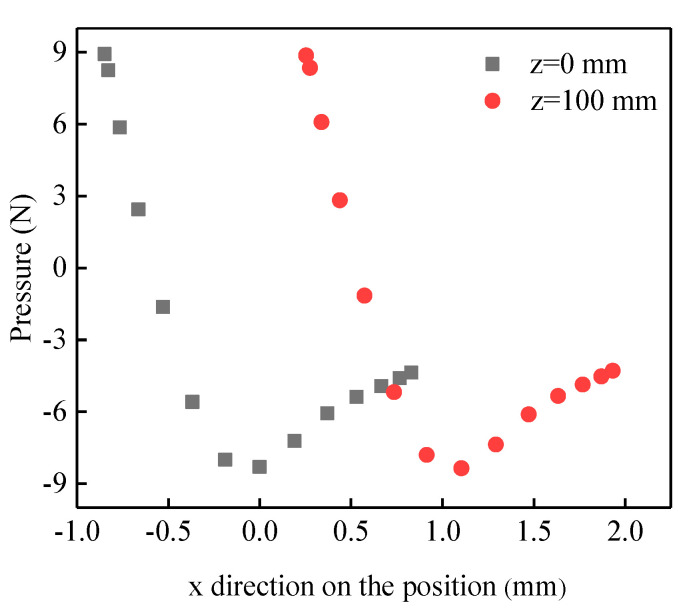
Scatter plot of pressure variation with the position in x-direction at z = 0 section and z = 100 section position.

With the change of the length in air velocity, the velocity vector and pressure distribution area of the fluid flow do not change; only the corresponding values are changed. Therefore, the pressure distribution cloud diagram and flow field velocity vector distribution cloud diagram obtained by a two-way fluid-structure interaction at the membrane length of 200 mm at the air velocity of (t = 1 s) 3 m/s are given under different lengths and different air velocities. As shown in Figure 8, the pressure value in the direction of the windward inlet is significantly greater than the pressure value in the direction of the leeward outlet. This is also the main reason for the fluid-induced vibration of the membrane tube. The velocity density near the wall of the windward inlet is increasing. The velocity gradient changes are increasing. The velocity of the fluid domain on the leeward side of the membrane tube is relatively lower than that of other regions. The data is processed for the pressure on the fluid-structure interaction contact surface. z = 0 cross-section and z = 100 cross-section of the pressure variation with the position in the x direction, as shown in Figure 9. It can be seen that the pressure value at the leftmost end of the contact surface is the largest, and the pressure value decreases along the x direction (airflow direction) and then increases in the opposite direction. The cross-sectional z = 100 position is shifted to the right to the z = 0 position. The flow field characterization shows a trend in vibration change consistent with the experiment.

## 5. Conclusions

This paper takes a single hollow fiber membrane as the research object, establishes the hollow fiber membrane of a two-way fluid-structure interaction model under the condition of airflow, and verifies the accuracy of the hollow fiber membrane in a two-way fluid-structure interaction model through experiments. The vibration and modal characteristics of single-root hollow fiber membranes with different lengths and air velocities are investigated by numerical simulation, and the flow characteristics are analyzed. The analysis leads to the following conclusions.

In the range of air velocity 1.5 m/s~6 m/s and membrane length 100~400 mm, the natural frequency of the hollow fiber membrane is negatively correlated with its length, and positively correlated with air velocity; compared with the change in air velocity, the natural frequency of each order is more sensitive than the change in length. The trend in higher order frequency variation with length is greater than that of lower order frequency variation. With the increase in the order, the dramatic area of the hollow fiber membrane mode increases, and the maximum deformation creeps from the middle to both ends.

The maximum deformation of the hollow fiber membrane occurs in the middle position, and the maximum equivalent stress is concentrated at both ends. The long time fixed at both ends can easily cause stress concentration, which may affect the life of the membrane. The maximum deformation and equivalent stress of the membrane increase with the increase in length and air velocity. The longer the length and the higher the air velocity, the more obvious the effect. The deformation and stress distribution caused by membrane tube vibration can be predicted based on fluid-structure interaction. The results can guide the design of membrane components.

Through the analysis of the pressure field and velocity field flow, the causes of hollow fiber membrane vibration are shown. The hollow fiber membrane vibration is mainly in the direction of airflow, which is consistent with the experimental law of the laser vibrometer.

## Figures and Tables

**Table 1:** Physical properties parameters.

Parameter	Value	Unit
Air density	1.23	kg/m^3^
Aerodynamic viscosity	1.89 × 10^−5^	kg/m·s^−1^
Membrane material density	1610	kg/m^−3^
Membrane Young’s modulus	280	MPa
Membrane Poisson’s ratio	0.38	-
Fiber outer diameter	1.7	mm
Fiber inner diameter	1.5	mm
Fiber length	100	mm

**Table 2:** Mesh independence verification.

Mesh Number	Maximum Deformation (mm)	Maximum Equivalent Stress (MPa)
55,110	0.0428	0.0526
108,300	0.0433	0.0536
433,000	0.0434	0.0538

**Table 3:** Experimental measurements and simulation results of hollow fiber membrane amplitudes in x and y directions at different air velocities.

Air Velocity (m^2^/s)	Amplitude in the x Direction (mm)			Amplitude in the y Direction (mm)		
	Experimental (mm)	Numerical (mm)	Deviation (%)	Experimental (mm)	Numerical (mm)	Deviation (%)
1.5	0.0266	0.027	1.51	0.0446	0.047	5.38
6	0.312	0.32	2.56	0.0226	0.023	1.76
10.5	0.758	0.76	0.26	0.051	0.05	−1.96

**Table 4:** The first 6-order natural frequencies with different fiber lengths at air velocity 1.5 m/s.

Frequency (Hz)	Air Velocity (1.5 m/s)			
	100 (mm)	200 (mm)	300 (mm)	400 (mm)
*f*_1_	83.94	22.09	11.95	5.23
*f*_2_	83.98	28.53	15.82	7.26
*f*_3_	230.15	60.01	29.54	14.48
*f*_4_	230.16	60.01	29.54	14.50
*f*_5_	448.1	117.63	54.74	28.37
*f*_6_	448.1	117.77	54.74	28.41

**Table 5:** The first 6-order natural frequencies with different fiber lengths at air velocity 3 m/s.

Frequency (Hz)	Air Velocity (3 m/s)			
	100 (mm)	200 (mm)	300 (mm)	400 (mm)
*f*_1_	84.01	24.78	12.47	7.37
*f*_2_	84.17	30.82	16.93	9.93
*f*_3_	230.24	63.23	30.35	16.30
*f*_4_	230.24	63.23	30.35	16.30
*f*_5_	448.19	119.27	55.69	30.31
*f*_6_	448.21	119.8	56.05	31.20

**Table 6:** The first 6-order natural frequencies with different fiber lengths at air velocity 4.5 m/s.

Frequency (Hz)	Air Velocity (4.5 m/s)			
	100 (mm)	200 (mm)	300 (mm)	400 (mm)
*f*_1_	84.29	26.10	12.77	8.25
*f*_2_	85.01	33.60	17.44	11.95
*f*_3_	230.62	65.06	30.83	20.78
*f*_4_	230.62	65.07	30.83	20.30
*f*_5_	448.61	121.37	56.26	35.89
*f*_6_	448.66	121.92	56.29	36.17

**Table 7:** The first 6-order natural frequencies with different fiber lengths at air velocity 6 m/s.

Frequency (Hz)	Air Velocity (6 m/s)			
	100 (mm)	200 (mm)	300 (mm)	400 (mm)
*f*_1_	84.97	26.57	13.41	9.48
*f*_2_	87.04	34.82	18.90	14.39
*f*_3_	231.54	65.06	31.86	21.17
*f*_4_	231.56	65.94	31.86	21.17
*f*_5_	449.63	121.94	57.49	36.38
*f*_6_	449.8	122.77	57.72	36.61

## Data Availability

Not applicable.
